# Tumor-derived exosomes in hypoxic microenvironment: release mechanism, biological function and clinical application

**DOI:** 10.7150/jca.69278

**Published:** 2022-03-14

**Authors:** Da Qian, Yaoyao Xie, Mingyao Huang, Jianfeng Gu

**Affiliations:** 1 Department of Burn and Plastic Surgery-Hand Surgery, First People's Hospital of Changshu City, Changshu Hospital Affiliated to Soochow University, Soochow, China.; 2 College of Pharmaceutical Science, Soochow University, Soochow, China.; 3 Department of General Surgery, The Second Affiliated Hospital of Fujian Medical University, Quanzhou, China.; 4 Department of General Surgery, First People's Hospital of Changshu City, Changshu Hospital Affiliated to Soochow University, Soochow, China.

**Keywords:** Hypoxic microenvironment, Cancer, Exosomes, Biological functions, Cancer therapy

## Abstract

Hypoxia is a key feature of solid tumors and is related to disease aggressiveness and adverse outcomes. It is recognized that the two-way communication between cancer cells and their microenvironment is critical to cancer progression. Increasing evidences show that the cellular communication and crosstalk between tumor cells and their microenvironment is not limited to secreted molecules, but also includes exosomes secreted by tumor cells. Exosomes are nano-scale extracellular vesicles (30-100 nm in diameter), which carry the molecular characteristics and cargo of the source cell, participating in intercellular communication through autocrine, paracrine and near-crine pathways. Recent studies have shown that cancer cells produce more exosomes under hypoxic conditions than normoxia conditions. The secretion and function of exosomes could be influenced by hypoxia in various types of cancer. Therefore, in this review, we summarize and discuss the latest research on the physiological mechanism of hypoxia regulating the secretion of exosomes, and the involvement of hypoxic exosomes in cancer progression and immune escape processes, and expounds the potential for targeting hypoxia-induced exosomes for cancer therapy strategies.

## Background

The initial research on hypoxia of cancers mostly comes from the judgment of the oxygen tension of cancer cells. In 1964, Cater used oxygen cathode technique to detect the difference of oxygen tension in cancer tissue 1. After that, in 1977 Vaupel named “Hypoxia in neoplastic tissue” for the first time and proved experimentally that with the increase in cancer volume, hypoxemia will appear in the cancer tissue, which may cause glucose depletion and cell lysis and necrosis 2.

Current research believes that cancer cells are exposed to a continuous medium of oxygen concentration, consisting of three tissue areas: normoxic zone, hypoxic zone, and necrotic zone 3. Normal oxygen cells are located near functional blood vessels and have the typical ability to survive and proliferate. Hypoxia usually occurs in solid cancers about 100 μm away from functional blood vessels 4. Cells about 150 μm away from blood vessels may be necrotic at very low oxygen concentrations (PO_2_≤1%) 5. Later, researchers find that cancer hypoxia has different characteristics that can be divided into acute hypoxia or chronic hypoxia 6, 7. The acute hypoxia, also known as ischemic hypoxia, is usually a structural and functional abnormality caused by the transient cancer disorder vasculature and structure 7, 8. Chronic hypoxia limited hypoxia is due to the imbalance between the supply and demand of oxygen as the distance of cancer growth and diffusion increases, and the rapid expansion of the cancer leads to insufficient oxygen in cancer tissue 70-150 μm from the open blood vessel 6. Acute and chronic hypoxia are associated with poor prognosis and cancer aggressive phenotype 8, 9.

The expression of most factors involved in the response of tumor cells to hypoxia is mainly regulated by hypoxia-inducible factor-1 (HIF-1). However, several other HIF-1 independent pathways, such as phospholipid protein trikinase (PI3K)-Akt, mammalian target rapamycin (mTOR), Wnt/β-caterine, mitochondrial activated protein kinase (MAPK), nuclear Factor-β and NADPH oxidase (NOX) are also involved in the adaptation of cancer cells under hypoxic conditions 10. It is recognized that the two-way communication between cancer cells and their microenvironment is critical to cancer progression. More and more evidences show that the cellular communication and crosstalk between hypoxic tumor cells and their microenvironment is not limited to secreted molecules, but also includes exosomes secreted by hypoxic tumor cells 11.

Exosomes are nano-scale extracellular vesicles (30-100 nm in diameter), which are the extracellular form of intraluminal vesicles (ILVs) secreted after multivesicular endosomes (MVEs) is injected into the plasma membrane 12. Exosomes released by different types of tumor cells carry the molecular characteristics and cargo of the source cell, and participate in intercellular communication through autocrine, paracrine and near-crine pathways 13, 14. The results of recent studies clearly show that the hypoxic environment strongly regulates the biogenesis of exosomes and participates in the progression of cancer. Therefore, in this review, we summarize and discuss the latest research on the physiological mechanism of hypoxia regulating the secretion of exosomes, and the involvement of hypoxic exosomes in cancer progression and immune escape processes, and expounds the potential for targeting hypoxia-induce exosomes for cancer therapy strategies.

## Response and adaptation to hypoxia in cancers

Hypoxia-induced changes in the proteome stimulate cancer growth, invasion, and metastasis by promoting adaptation and survival in harsh nutritionally deficient environments 8. At the molecular level, the adaptation of cancer cells to hypoxic stress is mainly regulated by HIF, which is a transcription factor that accumulates when cellular oxygen levels decrease 15, 16.

HIFs are members of the Arnt sim transcription factor superfamily, which consists of an heterodimer of oxygen sensitive α subunit and a constitutively expressed β subunit (HIF1 β) 17. At present, it has been found that there are three isoforms of HIF-1α, HIF-2α and HIF-3α 18. Under normal oxygen, HIFα protein was rapidly hydroxylated by a group of prolyl hydroxylase domain (PHD) enzymes, resulting in rapid degradation of HIF α 19. In the second mode of HIF α regulation, HIF α asparagine residues inactivate HIF α transcription activity by blocking the interaction of transcription cofactor cyclic adenosine monophosphate (cAMP) response element binding protein (CREB) binding protein (CBP) and histone acetyltransferase P300 (P300 hat) with HIF α through the inhibitory factor of HIF1 (FIH1; also known as HIF1AN), thereby blocking transcription 20. On the contrary, in the process of hypoxia, HIF α subunit did not hydroxylate, PHDs and FIH1 reduced the rate of HIFα protein hydroxylation, resulting in protein stabilization and CBP-p300 co-activation complex enhanced transcription activation, and increased HIFα level and HIF target gene expression activation 21.

## The biology of exosomes

Exosomes are ILVs formed by the inward budding of the endosomal membrane during the maturation of MVEs, which are nanoscale extracellular vesicles (30-100nm in diameter) secreted after the infusion of MVE with cell membrane. In the mid-1990s, it was reported that exosomes are secreted by B lymphocytes 22 and dendritic cells 23 with potential functions related to immune regulation and were considered to be vehicles in anti-tumor immune responses. Exosome secretion has now expanded to many different cell types, and its effect on cell-to-cell communication under normal and pathological conditions has now been fully demonstrated 24.

### Biogenesis of exosomes

Exosomes are generated in a process that involves double invagination of the plasma membrane. The first invagination of the plasma membrane forms the de novo formation of an early-sorting endosome (ESE), through the endocytosis of cell-surface proteins and soluble proteins associated with the extracellular milieu. ESE could merge with the endoplasmic reticulum (ER), trans-Golgi network (TGN), and may directly merge with a preexisting ESE in some cases 25-28. The further maturation of ESEs forms late-sorting endosomes (LSEs), and the invagination of the plasma membrane of LSEs forms ILVs that are ultimately secreted as exosomes with a size range of about 40 to 160 nm in diameter. In the end, the fate of MVEs is to be dissolved by fusion with autophagosomes or lysosomes, or to release exosomes by fusion with the plasma membrane 29.

### Machineries involved in the biogenesis of exosomes

Exosomes are generated within the endosomal system as ILVs during the process of maturation into MVE, a process that involves particular sorting machineries. At first, cargoes are segregated on the microdomains of the limiting membrane of MVE by these machineries, and then this microdomains inward budding and fission to form the small membrane vesicles containing sequestered cytosol 26.

In this process, the sorting of transmembrane cargoes into exosomes largely depends on the sorting machineries of endosomes. The discovery of endosomal sorting complexes required for transport (ESCRT) was a major breakthrough 30. Exosomes can also be formed in an ESCRT independent manner. Additonally, some other mechanisms also play an important role in promoting exosomes biogenesis. For example, chaperone heat shock 70kDa protein (HSP70) and heat shock homologous 71kDa protein (HSC71) can participate in the co-sorting of loading cytoplasmic proteins into ILVs 31. Some other transmembrane cargoes, such as glycosylphosphatidylinositol (GPI) anchored proteins, which probably enter exosomes due to their affinity for lipid domains and lipid rafts, directly participating in the generation of ILVs by affecting membrane-mediated biophysical properties. Additionally, the effects of ubiquitination 32 and farnesylation 33 have also been reported, but the concrete mechanisms are still not unclear.

Apart from proteins, exosomes can also load nucleic acids, including RNA (mRNA, miRNA, lncRNA) and DNA. Importantly, the selective loading of miRNA depends on its specific motif. Heterogeneous ribonucleoprotein A2B1(hnRNPA2B1) is a commonly expressed RNA-binding protein that controls the transport and subcellular localization of specific mRNA in neurons 34. The glycosylation of hnRNPA2B1 can recognize and bind to specific motifs on miRNA, thereby regulating miRNA entry into exosomes 35.

## The release of exosomes

### Transport of MVEs

After the formation of MVEs, MVEs have two fates. One is to be transported to lysosomes or autophagosomes to be degraded. The other one is to release exosomes after fusion with the plasma membrane. However, the results of these two methods are quite different. There is a potential balance mechanism between these two pathways. The establishment of this balance will undoubtedly affect the function of the cell, and its mechanism details have yet to be explored.

In general, intracellular transport involves organelles and cytoskeleton (actin and microtubules), related molecular motors (Dynein, kinetin and inosine) and molecular switches (small GTPases) 36, 37. Among them, small GTPases play a vital role in the transportation of MVEs. The Rab protein family is the largest subfamily in the small GTPases family. Of the 93 small GTPases members in Arabidopsis, 57 belong to the Rab subfamily 38. Different Rab proteins are located at specific locations on the cytoplasmic side of the plasma membrane, mainly using as molecular switches to regulate complex vesicle transport and microtubule system activity to regulate the transport of eukaryotic cell vesicle and protein. There are two different conformations between which Rab protein switches: GTP-bound active form and the other GDP-bound inactive form. In the activated form of GTP-bound, Rab can recruit some specific effector proteins to regulate vesicle formation and actin- and tubulin-dependent vesicle movement 39. Rab6 was initially considered to be a key participant in the retrograde transport of microtubules, controlling the transport of early vesicles from early endosomes through Golgi to TGN and from Golgi to the endoplasmic reticulum 40. It seems to be involved in a complex network of protein-protein interactions.

In addition, Rab27a and Rab27b and their respective effectors, synaptotagmin-like protein-4 and exophilin-5, are also essential for exosome secretion 41. Rab27b regulates the movement of MVEs torwards the plasma membrane, and both of these Rab27 isoforms can play a role in the step after MVEs are transported, which is the docking at the plasma membrane to promote fusion, and then increasing exosome secretion. Rearrangement of the sub-membrane actin cytoskeleton is a step that is common in all mechanisms involved in vesicle secretion. Naturally, the role of Rab27a in the docking of MVEs is also related to it 42. Additionally, KIBRA may act as an adaptor-like protein and stabilizer for Rab27a to prevent the degradation of Rab27a by ubiquitination, thereby promoting the secretion of exosomes 43. Rab27a can also control the secretion of secreted lysosomes (so-called lysosome-associated organelles), suggesting that MVE with exosome secretion capacity may be considered as a specialized compartment rather than a simple MVE subtype 44. Researchers also found that Rab11 uses Munc13-4 (Rab binding protein) as an effector to regulate the release of exosomes by regulating the docking of MVEs to the plasma membrane 45; Rab35 is localized in oligodendrocytes in a GTP-dependent manner to increases the density of vesicles, which may increase the secretion of exosomes through its docking or binding function 46.

These studies show that Rab subtypes are not constitutively expressed in all cell types, which means that each cell type can adapt to its own secretion mechanism for exosome secretion. Some other Rab subtypes, including Rab5, Rab7, and Rab22a, have also been reported in the role of MVEs transport.

### Fusion of MVEs with the plasma membrane

The final step in exosome secretion requires the fusion of MVEs with the plasma membrane to release exosomes as ILVs. This process may be mediated by members of the soluble N-ethylmaleimide sensitive factor attachment protein receptor (SNARE) 47 protein and synapse binding protein family 48. It is known that the SNARE complex involved in conventional lysosomal exocytosis is composed of vesicle-associated membrane protein 7 (VAMP7) on the lysosome, syntaxin 7 on the plasma membrane and lysosomal regulatory protein synapse binding protein 7. This complex is involved in exosome secretion 49 of certain cells (human leukemia cell line K562, but not in other cells (MDCK cells) 50. Exosome secretion process has been shown to be regulated by Ca2^+^ in several cell types, which may play a role in the activation of SNARE complex 51. Interestingly, synaptosome-related protein 23 (SNAP23) can regulate both exosome secretion 52 and lysosomal related organelles 53, which further strengthens the concept that MVE is a specialized secretory organelle. In addition, syntaxin 6 54 in prostate cancer; synaptic homologue Ykt6 55 in Drosophila; syntaxin 5 56 in C. elegans; and syntaxin 3 in human cytomegalovirus 47 all play a regulatory role in the secretion of exosomes, which again certificates that exosome regulatory factors may show diversity due to the differences in organisms, cell types, or MVE subtypes.

## The role of hypoxia in the regulation of exosome secretion

### The role of hypoxia in the sorting process

A variety of signaling molecules in the microenvironment can affect the level of ceramide. Hypoxia and ischemia can activate various signaling molecules to induce an increase in ceramide levels, which may be achieved by reducing the activity of ceramide enzymes 57. Accordingly, hypoxia can promote the biogenesis of ILVs, increasing the secretion of exosomes. In addition to the sorting mechanism and RNA binding protein, selective loading of miRNA in exosomes is also regulated by the external microenvironment. It has been reported that the level of miRNA detected in exosomes under hypoxia was significantly higher than that under normoxia, suggesting that hypoxia may have an effect on the distribution of miRNAs in exosomes, and this process may be carried out in a HIF-dependent manner 58. Among various cell types, microRNA-210 is considered to be one of the most stable and most important up-regulated miRNAs in response to hypoxia. The HRE on the proximal promoter of miR-210 can directly bind to HIF-1α and be regulated by it 59. In ovarian cancer, HIF can induce the release of exosomes rich in various miRNAs, including miR-21-3p, miR-125b-5p and miR-181d-5p 60. Similarly, under hypoxic conditions, miR-135b and miR-21 in exosomes secreted by melanoma and PANC cells also showed an increase 61, 62. Therefore, hypoxia may affect the distribution of miRNA in exosome by depending on HIF. However, the specific mechanisms still need to be further explored.

All in all, the biogenesis of exosomes is certainly complex; it depends on the cargoes and cell type.

### The role of hypoxia in the transport of MVEs

It has been shown that hypoxia can regulate the expression of Rab7 and Rab27a by inducing the expression of STAT3, thereby promoting the release of ovarian cancer cell exosomes 63; HIF can activate the transcription genes of Rab22a and small GTPase Rab22a expression mediates the release of breast cancer cell exosomes 64. Moreover, Rab5 has a possible mechanism of increased exosome secretion mediated in hypoxic PCa cells. It may regulate the transport of clathrin-coated vesicles from the cell membrane to early endosomes and the fusion of homotypic early endosomes, regulating the release of exosomes, and Rab5 accumulates more in the perinuclear area under hypoxic conditions 65. It means that Rab5 can regulate the possibility of exosome secretion in a hypoxic environment. Besides, hypoxia can also affect the expression of RHO-related protein kinase (ROCK) to regulate the rearrangement of the actin cytoskeleton, which in turn affects the release process of exosomes 66.

In addition to promoting the secretion of exosomes by regulating the transport process of MVEs, hypoxia has also been shown to promote autophagy 67. Therefore, we can speculate that the balance mechanism between the degradation of MVEs and the release of exosomes still exists in the hypoxic external environment, which need to be explored further.

**Figure 1:**
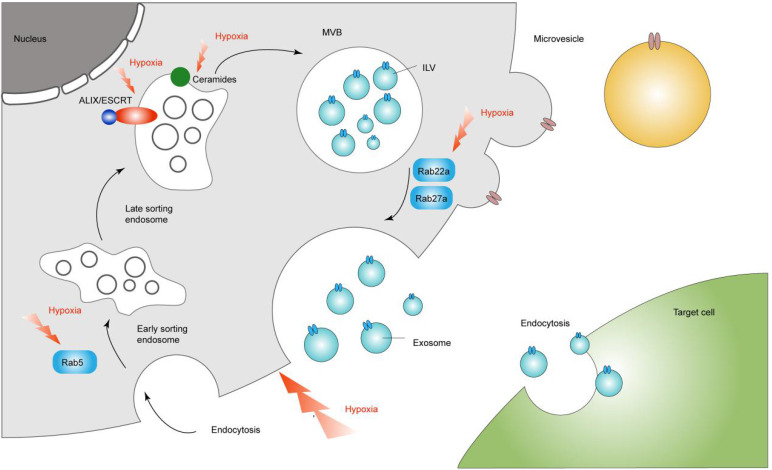
Hypoxia in the regulation of exosome secretion. Hypoxia regulates exosomal secretion by affecting processes such as Rab5, Rab22a, Rab27a, ceremides and ALIX/ESCRT.

As mentioned in the review, we can recognize the regulatory role of hypoxia in the process of exosome secretion (Figure 1). This gives us a new direction for targeted treatment of tumors through the regulation of exosomes through the hypoxic microenvironment.

## The role of hypoxia-induced exosomes involved in tumor microenvironment

Increasing studies have found that exosomes are involved in the formation and metastasis of cancer blood vessels, and lead to the immune escape of cancer by regulating the differentiation of immune cells (Table 1).

### Hypoxia-induced exosomes promote cancer angiogenesis and metastasis

According to conventional opinion, cells enter the bloodstream from the primary tumor. They then implant to distant organs like the lungs, where the cells grow into metastatic tumors. Another idea is that the primary tumor sends out exosomes, which prepare the tumor cell to take root at a distance and recruit nourish incoming tumor cell. It is reported that lncRNA-ROR can promote the growth of hepatocellular carcinoma cells under hypoxic stress 68. Similarly, exosomes, as mediators of cell-to-cell communication, have also been reported to be involved in the angiogenesis and metastasis of cancer under hypoxic stress. For example, exosomes miR-1273f and miR-23a/b of hypoxic HCC cells promote the aggressive phenotype of cancer cells and drive the growth and development of HCC 69, 70; Interestingly, exosomes released from HCC under hypoxia have also been found to be able to enrich miR-155. Exosomes increase miR-155 in HUVECs, thereby promoting the formation of blood vessels 71. The renewal of blood vessels contributes to tumor metastasis. Therefore, we further explored the impact of exosomes released by tumors on tumor metastasis in the hypoxic microenvironment.

A recent study showed that hypoxic oral squamous cell carcinoma (OSCC) promotes the secretion of exosomes through HIF-1 and HIF-2, and the miR-21 carried by exosomes can promote the migration and invasion of other oral squamous cell carcinoma cells 58. Meanwhile, the researchers observed the internalization process of bladder cancer cells with exosomes by fluorescently labeling exosomes. At the same time, they detected a significant high expression of lncRNA-UCA1 in cells with internalized exosomes. Further *in vitro* experiments confirmed that exosomes promote the EMT process, which indicating that hypoxia exosome lncRNA-UCA1 of bladder cancer cells promoted the invasive phenotype. Importantly, *in vivo* experiments also confirmed that the exosomes released by hypoxic bladder cancer cells can promote the proliferation of bladder cancer more significantly than that of normoxic bladder cancer cells 72.

### Hypoxia-induced exosomes influence cancer immune escape

Increasing evidences show that tumor-derived exosomes can induce T cell apoptosis, reduce NK cell activity, inhibit IFN-γ-dependent type II macrophage expression, and change the differentiation of monocytes to increase the number of bone marrow-derived suppressor cells (MSDC), which reduces immune surveillance and thus causes immune escape from tumors 72-74.

### T cells

With the deepening in our understanding of the pathogenesis and characteristics of cancer, the importance of the immune system in tumor progression has been generally recognized. A large number of studies have shown that the exhaustion of CD8^+^ T cells and the expansion of Tregs in tumor-infiltrating lymphocytes play an important role in tumor progression and immune escape 75. It is reported that exosomes derived from gastric cancer cells change CD8^+^T cell gene expression, thereby inducing CD8^+^T cell apoptosis 76. A recent study also found that exosomal miR-208b related with Oxaliplatin resistance promotes Tregs expansion in colorectal cancer 77. It is worth noting that the role of exosomes released by cancer in the regulation of T cell differentiation under hypoxic microenvironment cannot be ignored. Given that hypoxia is a common feature of solid tumors and alter tumor exosome levels, Ling Li et al. found that tumor exosomes alter the proliferation and cytotoxicity of T cells in a way that relies on HSP70 but not dendritic cells. Further studies have found that exosomal miR-21 from OSCC enhance the inhibitory effect of MDSCs on T cells through the PTEN/PD-L1 axis, thereby causing cancer progression 78. Additionally, Ye et al 79. isolated exosomes from nasopharyngeal carcinoma (NPC) cell lines and patient sera, or control NP69 cells and healthy donor sera and found that miR-24-3p was markedly enriched in cancer derived exosomes. The study revealed that exosomes derived from tumor hypoxic cells mediate the transfer of miR-24-3p from tumor cells to T cells, inhibiting the proliferation of Th1 and Th17, while inducing the differentiation of Tregs. The future challenge will be to combine bioengineering technology to realize the application of exosomes released by hypoxic tumors in immunotherapy.

### Macrophages

Since macrophages are involved in innate immunity, they are an integral part of the tumor microenvironment. Tumor-associated macrophages (TAM) play a major role in cancer-related inflammation and constitute an important regulator of tumorigenesis 80. According to different phenotypes, TAMs are usually divided into two categories: M1-like and M2-like 81. M1-TAM is mainly involved in the inhibition of tumor growth 82. In contrast, M2-TAMs mainly exert immunosuppressive function and promote tumor growth 83. A recent study showed that pancreatic cancer cell derived hypoxia exosomes express miR-301a-3p can polarize macrophages through the PTEN/PI3K signaling pathway. The polarization of macrophages leads to enhanced metastasis potential of pancreatic cancer cells *in vitro* and *in vivo*. Therefore, this study considered exosome miR-301a-3p as a new target for mediating tumor immune avoidance 84. In addition, extracellular vesicles from hypoxic lung cancer also increased the M2 polarization of macrophages through the transfer of miR-103a. The mechanism of this process that hypoxic exosome miR-103a increases the activation of AKT and STAT3 and expression of angiogenic factors 85. Interestingly, another study found that hypoxia induces macrophage polarization involving the expression of exosomes in epithelial ovarian cancer 86. Specifically, the study showed that SKOv3 cells and their secreted exosomes under hypoxic conditions expressed much more miR-940 than cells and exosomes under normoxic conditions. Importantly, the researchers also found that cancer-derived exosomes in a hypoxic microenvironment can significantly increase the expression of M2-Like macrophage marker proteins, which suggested that exosomes derived from epithelial ovarian cancer could deliver miR-940 to macrophages, thereby inducing macrophages to an M2-like phenotype. The above studies indicate that under hypoxic microenvironment, cancer promotes the polarization of macrophages to M2 by secreting exosomes, thereby shaping an inhibitory tumor microenvironment.

### Natural killer cells

In recent years, the role of the innate immune system in anti-tumor therapy has received increasing attention due to its possible role in the early stages of tumor development 87. As a subset of intact congenital lymphoid cells, NK cells are thought to be capable of killing primary tumor cells and metastatic cells by producing natural toxicity and performing functions similar to CD8^+^ cytotoxic T cells 88. Interestingly, some studies have also found that NK cells can be regulated by exosomes. It is reported that exosomes secreted by HCC can carry circUHRF1, inhibit miR-449c-5p through the sponge effect, thereby up-regulating the expression of T cell immunoglobulin and mucin domain 3 (TIM-3). Tim-3 exerts its immunosuppressive effect, causing the immune escape of HCC 89. Coincidentally, Szczepanski et al. found that exosomes from acute myeloid leukemia can down-regulate the expression of NK cell activation receptors, especially NKG2D. This process is induced by tumor-derived exosomes carrying the TGF-β 90. It is worthy of our attention that hypoxic stress can also participate in the regulation of NK cells by exosomes. Burchem et al. cultivated lung cancer cells by establishing a hypoxic microenvironment *in vitro*, and found that hypoxia promoted the secretion of MVs-TGF-β by lung cancer cells, thereby impairing NK-mediated cytotoxicity and NK cell function 91. Further research also found that the impairment of NK-mediated cytotoxicity by hypoxic tumor-derived MVs involves a decrease in NKG2D induced by TGF-β. This mechanism is consistent with the study by Szczepanski et al under normoxia. This result indicates that the hypoxic microenvironment mainly plays a role in promoting the release of extracellular vesicles, and exerts an inhibitory effect on NK cells through the increase of extracellular vesicles.

**Figure 2:**
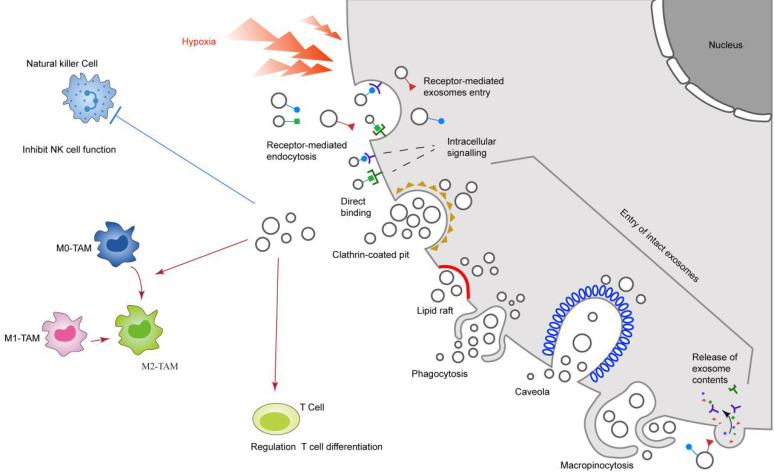
Hypoxia-induced exosomes influence cancer immune system. The solid red line represents the promoting effect, and the dotted blue line represents the inhibitory effect. Hypoxia promotes the release of exosomes from cancer cells, thereby inhibiting the function of NK cells and inducing the differentiation of macrophages and T cells.

In short, exosomes can act as biologically active vesicles, regulating the functions of different types of immune cells by participating in more than one molecular pathway responsible for the genetic changes of recipient cells (Figure 2).

## The potential application of hypoxia-exosomes in cancer treatment

In view of the important role of exosomes in regulating the immune microenvironment, depleting exosomes or blocking the uptake of exosomes may become a new cancer immunotherapy 25. In recent years, a new device that can remove blood components below 200 nm, including tumor-derived exosomes that interact with the fixed affinity agent of the device, called the Aethlon ADAPT™ system, has been successfully applied to hepatitis C for the first time Virus patients 92. Studies have found that hypoxia-induced exosomal circZNF91 can competitively bind miR-23b-3p when delivered to normoxic pancreatic cancer cells, thereby eliminating the inhibition of miR-23b-3p on the expression of deacetylase Sirtuin1 (SIRT1). Therefore, up-regulated SIRT1 enhances the deacetylation-dependent stability of HIF-1α protein, and promotes the Gemcitabine resistance of normoxic pancreatic cancer cells 93. In addition, the study also found that hypoxic tumour cell-derived exosomal miR-340-5p promotes radioresistance of oesophageal squamous cell carcinoma 94. It could be speculated that if the Aethlon ADAPT™ system were used to eliminate exosomal circZNF91 or miR-340-5p, it may improve the efficacy of Gitacitabine and radiotherapy. In addition, the development of inhibitors for the exosome release pathway is also a promising treatment strategy. For example, histone deacetylase 6 (HDAC6), a tumor suppressor, restores the phagocytosis of macrophages by inhibiting the exosomal miRNA let-7i-5p, thereby inhibiting tumor progression 95. However, these studies are currently only based on animal experiments, and there are no studies on patients. In the future, further clinical trials are needed to explore the clinical application of exosomes.

## Conclusions and perspectives

The regulation of the release and function of exosomes by hypoxia is now considered a new and exciting field in cancer research. Revealing the mechanism of hypoxia regulating exosomes secretion and the biological functions of exosomes will help to find targets for intervention in exosomes secretion and strategies to treat cancer. Current studies have found that hypoxia regulates the secretion of exosomes by regulating the sorting process and transport of MVEs. This suggests that in the future, researchers can target these two processes to develop drugs for exosomal secretion, so as to suppress tumors at an early stage.

In addition, the above studies have shown that exosomes play a role in angiogenesis and tumor metastasis, and can cause tumor immune escape by regulating the differentiation of immune cells. This helps us understand the mechanism of hypoxic exosomes transfer and target cell selection, thereby improving the prospects of exosomal therapeutic targeting and its development as a therapeutic delivery vehicle. However, the technology for exosome targeting is not yet fully mature and needs to be further explored. As these unknown factors are gradually revealed, we believe that exosomes will become an important tool for cancer treatment in the near future.

## Figures and Tables

**Table 1:** The role of hypoxia-induced exosomes involved in cancer biology

Source cells	Regulatory factors	Biological function	Mechanism	Ref
Hepatocellular carcinoma	Exosomal miR-1273f	Increase angiogenesis	Downregulate its target LHX6	69
Hepatocellular carcinoma	Exosomal miR-23a/b	Increase angiogenesis	Target the von Hippel-Lindau/hypoxia-inducible factor axis	70
Hepatocellular carcinoma	Exosomal miR-155	Increase angiogenesis	/	71
Oral Squamous Cell Carcinoma	Exosomal miR-21	Increase the migration and invasion	HIF-1 and HIF-2 increase the expression of miR-21	58
Bladder cancer	Exosomal lncRNA-UCA1	Increase the migration and invasion	LncRNA-UCA1 promotes tumor progression though EMT	72
Oral Squamous Cell Carcinoma	Exosomal miR-21	Regulate immune response	Target PTEN/PD-L1 axis	78
Nasopharyngeal carcinoma	Exosomal miR-24-3p	Regulate immune response	Downregulate its target FGF11	79
Pancreatic cancer	Exosomal miR-301a-3p	Regulate immune response	Target PTEN/PI3K axis	84
Lung cancer	Exosomal miR-103a	Regulate immune response	Target AKT/STAT3 axis	85
Epithelial ovarian cancer	Exosomal miR-940	Regulate immune response	/	86
Lung cancer	Exosomal TGF-β	Regulate immune response	Decrease the expression of NKG2D	91

## Abbreviations

HIF-1: Hypoxia-inducible factor-1
PI3K: Phospholipid protein trikinase
MAPK: mitochondrial activated protein kinase
NOX: NADPH oxidase
PHD: Prolyl hydroxylase domain
VHL: Von Hippel Lindau
cAMP: cyclic adenosine monophosphate
CREB: cAMP response element binding protein
ILVs: intraluminal vesicles
MVE: multivesicular endosomes
ESE: early-sorting endosome
ER: endoplasmic reticulum
TGN: trans-Golgi network
ESCRT: endosomal sorting complexes required for transport
ALIX: ALG-2 interacting protein X
LBPA: lysobisphosphatidic acid
S1P: Sphingosine1-Phosphate
SNARE: soluble N-ethylmaleimide sensitive factor attachment protein receptor
HSP70: heat shock 70kDa protein
HSC71: heat shock homologous 71kDa protein
GPI: glycosylphosphatidylinositol
HnRNPA2B1: Heterogeneous ribonucleoprotein A2B1
SNAP23: Synaptosome-related protein 23
ROCK: RHO-related protein kinase
MSDC: marrow-derived suppressor cells
MSDC: marrow-derived suppressor cells
TAM: Tumor-associated macrophages
TIM-3: T cell immunoglobulin and mucin domain 3
NPC: nasopharyngeal carcinoma
SIRT1: Sirtuin1
HDAC6: Histone deacetylase 6
Gi: G protein
CBP: cAMP response element binding protein binding protein
VAMP7: vesicle-associated membrane protein 7
